# Pharmacodynamic comparison of different antimicrobial regimens against *Staphylococcus aureus* bloodstream infections with elevated vancomycin minimum inhibitory concentration

**DOI:** 10.1186/s12879-020-4782-9

**Published:** 2020-01-23

**Authors:** Thaina Miranda da Costa, Gabriel Trova Cuba, Priscylla Guimarães Migueres Morgado, David P. Nicolau, Simone Aranha Nouér, Kátia Regina Netto dos Santos, Carlos Roberto Veiga Kiffer

**Affiliations:** 1 Laboratório de Infecção Hospitalar, Departamento de Microbiologia Médica, Instituto de Microbiologia Paulo de Góes, Universidade Federal do Rio de Janeiro, Av. Carlos Chagas Filho, 373 - bloco I, Sala I2-010, Cidade Universitária Rio de Janeiro, Rio de Janeiro, Brazil; 2 Laboratório Especial de Microbiologia Clínica, Disciplina de Infectologia, Escola Paulista de Medicina, Universidade Federal de São Paulo (UNIFESP), Rua Leandro Dupret, São Paulo, SP 188 Brazil; 3 Center for Anti-infective Research and Development, Hartford Hospital, 80 Seymour Street, Hartford, CT USA; 4 Hospital Universitário Clementino Fraga FilhoFaculdade de Medicina, Universidade Federal do Rio de Janeiro, Rua Rodolpho Paulo Rocco, 255, Rio de Janeiro, RJ Brazil

**Keywords:** Bloodstream infections, *Staphylococcus aureus*, Pharmacodynamic targets, Ceftaroline, Daptomycin, Vancomycin

## Abstract

**Background:**

*Staphylococcus aureus* is one of the major causes of bloodstream infections (BSI) worldwide, representing a major challenge for public health due to its resistance profile. Higher vancomycin minimum inhibitory concentrations (MIC) in *S. aureus* are associated with treatment failure and defining optimal empiric options for BSIs in settings where these isolates are prevalent is rather challenging. In silico pharmacodynamic models based on stochastic simulations (Monte Carlo) are important tools to estimate best antimicrobial regimens in different scenarios. We aimed to compare the pharmacodynamic profiles of different antimicrobials regimens for the treatment of *S. aureus* BSI in an environment with high vancomycin MIC.

**Methods:**

Steady-state drug area under the curve ratio to MIC (AUC/MIC) or the percent time above MIC (*f*T > MIC) were modeled using a 5000-patient Monte Carlo simulation to achieve pharmacodynamic exposures against 110 consecutive *S. aureus* isolates associated with BSI.

**Results:**

Cumulative fractions of response (CFRs) against all *S. aureus* isolates were 98% for ceftaroline; 79% and 92% for daptomycin 6 mg/kg q24h and for the high dose of 10 mg/kg q24h, respectively; 77% for linezolid 600 mg q12h when MIC was read according to CLSI M100-S26 instructions, and 64% when MIC was considered at the total growth inhibition; 65% and 86% for teicoplanin, three loading doses of 400 mg q12 h followed by 400 mg q24 h and for teicoplanin 400 mg q12 h, respectively; 61% and 76% for vancomycin 1000 mg q12 h and q8 h, respectively.

**Conclusions:**

Based on this model, ceftaroline and high-dose daptomycin regimens delivered best pharmacodynamic exposures against *S. aureus* BSIs. Teicoplanin higher dose regimen achieved the best CFR (86%) among glycopeptides, although optimal threshold was not achieved, and vancomycin performance was critically affected by the *S. aureus* vancomycin MIC ≥2 mg/L. Linezolid effectiveness (CFR of 73%) is also affected by high prevalence of isolates with linezolid MIC ≥2 mg/L. These data show the need to continually evaluate the pharmacodynamic profiles of antimicrobials for empiric treatment of these infections.

## Background

Bloodstream infections (BSI) are conditions of elevated incidence in nosocomial environments, particularly in critically ill patients. They have been associated with longer hospital stays, higher costs, and higher crude mortality rates [1]. *Staphylococcus aureus* is one of the main pathogens associated with BSI worldwide and in Brazil, and methicillin-resistant *S. aureus* (MRSA) is associated with a significant elevated mortality risk [1, 2]. Although *S. aureus* is a challenge for public health, vancomycin has been the cornerstone of treatment of patients with MRSA infections for more than fifty years [3, 4]. However, increasing reports of reduced susceptibility to vancomycin are of great concern, especially since higher mortality rates in MRSA BSI have been associated with increased vancomycin MIC (≥ 2 mg/L in some studies) [5, 6]. While several anti-MRSA agents such as ceftaroline, daptomycin and linezolid have been used as alternatives to glycopeptides (vancomycin and teicoplanin), limited clinical and pharmacodynamic (PD) comparative data are available to assess their utility in MRSA BSI [7–9].

Monte Carlo simulation is a stochastic prediction tool, which is implemented by computer mathematical methods associating different variables, such as the pharmacokinetic (PK) profile of antimicrobials and the in vitro susceptibility data (i.e., MIC distribution), in order to estimate the antibiotics dosing regimens’ probability of achieving targeted PD exposures [10]. The use of in silico PD models based on stochastic simulations is an important tool to explore optimal antimicrobial regimens in different populations or in specific settings [11, 12]. These models allow exploration of different dosing regimens and targets in virtual scenarios, in order to explore best strategies to overcome challenging conditions [11], such as resistance emergence. Previous evaluations of different antimicrobial PD against *S. aureus* have already been published [13, 14].

The aim of the present study was to determine the probability of target attainment of different regimens of ceftaroline, daptomycin, linezolid, teicoplanin, and vancomycin using Monte Carlo simulation. The probability of microbiological success of these antimicrobials based on PK/PD simulations was assessed against *S. aureus* isolates from BSI from an university teaching hospital, where resistance to various antimicrobials and increasing MIC values has been previously observed [15].

## Methods

### Microbiology and ethics statement

This study was performed at the University Hospital Clementino Fraga Filho, a public tertiary teaching hospital in Rio de Janeiro, Brazil, with about 70,000 patients/day per year during the study period. It was approved by Human Research Ethics Committee of the University Hospital Clementino Fraga Filho (number 008/15).

A retrospective analysis was conducted evaluating all episodes of *S. aureus* BSI occurring in adults between February 2011 and December 2013, as defined by hospital control specialists as the first *S. aureus* isolate from a BSI episode and with subsequent initiation of anti-staphylococcal therapy. Only single and consecutive isolates were included in the present study (i.e. one *S. aureus* isolate from a single patient with first episode submitted to therapy).

All blood cultures were routinely processed using BacT/ALERT® (BioMerieux Inc., Durham, NC, USA) during the referred period. Bacterial identification was carried out by the automated VITEK® 2 system (BioMerieux, Durham, NC, USA). Identification of bacteria was confirmed using Gram staining, catalase and coagulase production, and evaluation of 0.04 U bacitracin resistance by disk-diffusion [16].

Cefoxitin disk (CECON, São Paulo, Brazil) diffusion test for evaluating oxacillin resistance was performed for all selected isolates, according to CLSI recommendations [17]. Determination of MICs was performed by broth microdilution, using fresh cation-adjusted Mueller-Hinton broth (CAMHB) for vancomycin, teicoplanin, linezolid, daptomycin (Sigma-Aldrich Chemical Company, St Louis, MO, USA) and ceftaroline (donated by AstraZeneca Pharmaceuticals, Schaumburg, IL, USA). CAMHB was supplemented with 50 μg/mL calcium for daptomycin assay. Linezolid MIC was determined considering growth inhibition of 100% (linezolid-100) and also according to CLSI M100-S26 document (designated linezolid-80). The CLSI interpretative breakpoints were used for all antimicrobials [17]. The ATCC strains 25923 and 29213 were used as controls for the disk diffusion and MIC tests, respectively. The *mec*A gene detection was performed as previously described [18] for isolates resistant to cefoxitin by disk diffusion.

### Antibiotic regimens

Steady-state exposure was assessed for the following antibiotic regimens by the methodology described below: daptomycin 6 mg/kg q24h; daptomycin 10 mg/kg q24h; linezolid 600 mg q12h; teicoplanin three 400 mg q12h as loading dose, followed by 400 mg q24h; teicoplanin 400 mg q12h; vancomycin 1000 mg q8h; vancomycin 1000 mg q12h; ceftaroline 600 mg q12h.

### Pharmacokinetic / pharmacodynamic (PK/PD) models

Mean PK parameters and their distributions were extrapolated from published patient studies for each antibiotic. For studies to be considered, they had to be conducted in at least 10 actual patients (defined as a clinical study in the presence of an infection), to have described the assay used to determine drug concentrations and presented mean and standard deviation results for the total body clearance in liters per hour, volume of distribution of the central compartment and other pertinent PK parameters. Mean PK parameters and distribution were extrapolated from selected published studies for each antibiotic [19–22], and distribution curves were assumed in the PD model according to each parameter.

The PK/PD parameters (*f*AUC, total AUC or *f*T > MIC) were chosen based on PD exposure-response relationship for each agent [22–26]. AUC_0-24h_ was calculated by dividing Dose_24hours_/Clearance and then this value was divided by each MIC dilution between 0.625 mg/L to 16 mg/L to provide the total AUC/MIC (for linezolid and vancomycin) or *f*AUC/MIC (for daptomycin) calculation [13, 14]. Teicoplanin AUC_0–24_ was calculated using the trapezoidal rule and divided by each MIC dilution as previously described [13]. Each antimicrobial and their respective PK published studies and PD targets adopted are described in specific subsections (described in more detail below). Supplementary table summarizes the PK parameters derived from published studies, their respective references, and the PD targets chosen for each antimicrobial used in the Monte Carlo simulation.

### Daptomycin

The PK parameters of daptomycin were derived from a study with 58 subjects treated for severe gram-positive infections [19]. Daptomycin PK model considered an 80 kg weight individual, with total body clearance of 0.8 ± 0.14 L/h, protein binding of 90 to 93%, linear and dose-proportional PK over dose range studied (6 mg/kg/dose and 10 mg/kg/dose) [19, 27]. Daptomycin PD target of *f*AUC/MIC > 40 was chosen, previously associated with bacteriostasis in thigh murine infection model [23].

### Linezolid

Linezolid PK data was obtained from a study with 318 adults with gram-positive infections (community-acquired pneumonia and skin and soft tissue infections) treated under the compassionate-use protocol [20]. The Linezolid PK model then assumed a total body clearance of 6.85 ± 3.45 L/h [20] and a one-compartment model [13, 28]. Linezolid PD target was total AUC/MIC > 82.9, exposure required for a bacteriostatic response in neutropenic murine thigh infection model [24].

### Teicoplanin

Teicoplanin PK were derived from a population study with 30 febrile and severely neutropenic patients. Teicoplanin PK then assumed a total body clearance of 1.15 ± 0.56 L/h, volume of the central compartment (6.56 ± 4.01 L), k12 (1.29 ± 0.62 h)^− 1^) and k21 (0.18 ± 0.08 h)^− 1^), and a two-compartment model until steady state, to account for its long half-life [13, 21]. Teicoplanin PD target was a total AUC/ MIC ≥900, exposure correlated with bacteriological response in patients with documented MRSA infection [25].

### Vancomycin

Vancomycin PK data were derived from a populational study of patients receiving treatment for *S. aureus* lower respiratory tract infection [22]. Vancomycin total body clearance was estimated as a function of creatinine clearance (CrCl, mL/min); drug clearance (L / h) = [(CrCl · 0.79) + 15.4] · 0.06 [22]. CrCl was assumed to follow a triangular distribution, simulated as a range between 50 mL/min and 120 mL/min [13]. Vancomycin PD target was total AUC/ MIC ≥350, which was the exposure associated with clinical success for lower respiratory tract infections, approximately corresponding to a trough vancomycin concentration of 15–20 mg/mL [29].

### Ceftaroline

Ceftaroline probability of target attainment (PTA) was obtained from literature [26, 30]. The model was derived from a three-compartment model developed from plasma concentration from Phase 1, 2, 3 studies, the two latter in patients with complicated skin infection and community-acquired pneumonia [31]. Ceftaroline PD target used was 51% *f*T > MIC, which is an exposure associated with 2-log_10_ CFU reduction from baseline for *S. aureus* on murine thigh and lung infection models [26].

### Monte Carlo simulation

A 5000-patient Monte Carlo simulation (Crystal Ball 2000; Decisioneering Inc., Denver, CO, USA) was performed to calculate a population of total AUC/MIC (free or total) or T > MIC exposures for each antibiotic regimen at each MIC dilution. Clearance, volume of the central compartment, k12 and k21 were each assumed to follow log-Gaussian distributions during simulations for teicoplanin. For the vancomycin simulation, creatinine clearance was assumed to follow a triangular distribution as previously described [13]. The number of simulated patients achieving the target PD exposure at each MIC was counted and reported as the PTA at that specific MIC (values in percentages). The cumulative fractions of response (CFR) was calculated as previously described for each drug [32], multiplying the PTA at each MIC by the percentage of isolates with that specific MIC. Final CFR results were obtained as the sum of each PTA per MIC and a CFR ≥ 90% was considered optimal [14].

### Sensitivity analysis

A sensitivity analysis was conducted to explore the robustness of the CFR against entire BSI *S. aureus* isolates. In order to perform the analysis, different PD targets were applied as a way of comparison with targets originally applied, as follows. For vancomycin sensitivity analysis, an alternative PD target of AUC/MIC > 400 was used, which was the exposure associated with superior clinical response in lower respiratory tract infections [22]. For daptomycin, PD targets of *f*AUC/MIC > 12 (the minimum value providing static effect with MRSA) and > 171 (the minimum ratio which provided 99% kill) were obtained from a thigh murine infection model [23]. For linezolid, the alternative PD targets of total AUC/ MICs > 51.85 (minimum breakpoint associated with clinical cure) and > 128 (median AUC/MIC associated to bacterial eradication in the blood of adult patients enrolled in the compassionate use program of linezolid) were also explored [33]. For ceftaroline, *f*T > MIC targets of 26 and 36% were also analyzed, which are targets associated to bacterial reduction endpoints of net bacterial stasis and 1-log_10_ CFU reductions from baseline for *S. aureus* based on murine infection models, respectively [26]. Finally, teicoplanin trough value of > 13 mg/L and > 20 mg/L were investigated, they are currently used in clinical practice [34, 35].

## Results

A total of 110 single *S. aureus* isolates associate to one BSI episode were included in the analysis. It is relevant to note that 25 (23%) isolates presented MIC = 2 mg/L and 6 (5%) presented MIC = 4 mg/L to vancomycin, respectively. One VISA isolate was also ceftaroline-intermediate (MIC of 2 mg/L). Thirty-one (28%) isolates were resistant to cefoxitin by disk diffusion and carried the *mec*A gene (MRSA).

Table 1 summarizes the MIC distributions of vancomycin, teicoplanin, linezolid, daptomycin and ceftaroline. Both linezolid readings were considered as part of the exploratory model for CFR analysis. It was observed that linezolid MICs ranged from 0.25 to 4 mg/L and from 0.25 to 2 mg/L for linezolid-100 and-80, respectively. The MIC results for linezolid-80 were one doubling-dilution lower than those read at 100% inhibition for 39 (35%) isolates. Non-susceptibility to daptomycin was observed in 16 (14.5%) isolates, which represented 35.5% of the MRSA isolates.

**Table 1:** Minimum Inhibitory Concentrations distributions determined for 110 *Staphylococcus aureus* isolates from bloodstream infections

Antimicrobial	N° (%) of isolates						
	MIC value in mg/L						
	0.0625	0.125	0.25	0.5	1	2	4
Ceftaroline	2 (2)	51 (46)	33 (30)	11 (10)	12 (11)	1 (1)	0
Daptomycin	0	0	3 (3)	26 (23)	65 (59)	12 (11)	4 (4)
Linezolid-100^a^	0	0	1 (1)	0	15 (14)	83 (75)	11 (10)
Linezolid-80^a^	0	0	1 (1)	1 (1)	42 (38)	66 (60)	0
Teicoplanin	0	0	71 (64)	33 (30)	5 (5)	0	1 (1)
Vancomycin	0	0	0	4 (4)	75 (68)	25 (23)	6 (5)

*MIC* Minimum Inhibitory Concentrations, ^a^: *MIC* Endpoint values for linezolid were read at the first well where the trailing begins without regard for pinpoint trailing, as CLSI M100-S26 instructions, being designated as linezolid-80; and at 100% inhibition of growth, identified as linezolid-100

**Fig. 1:**
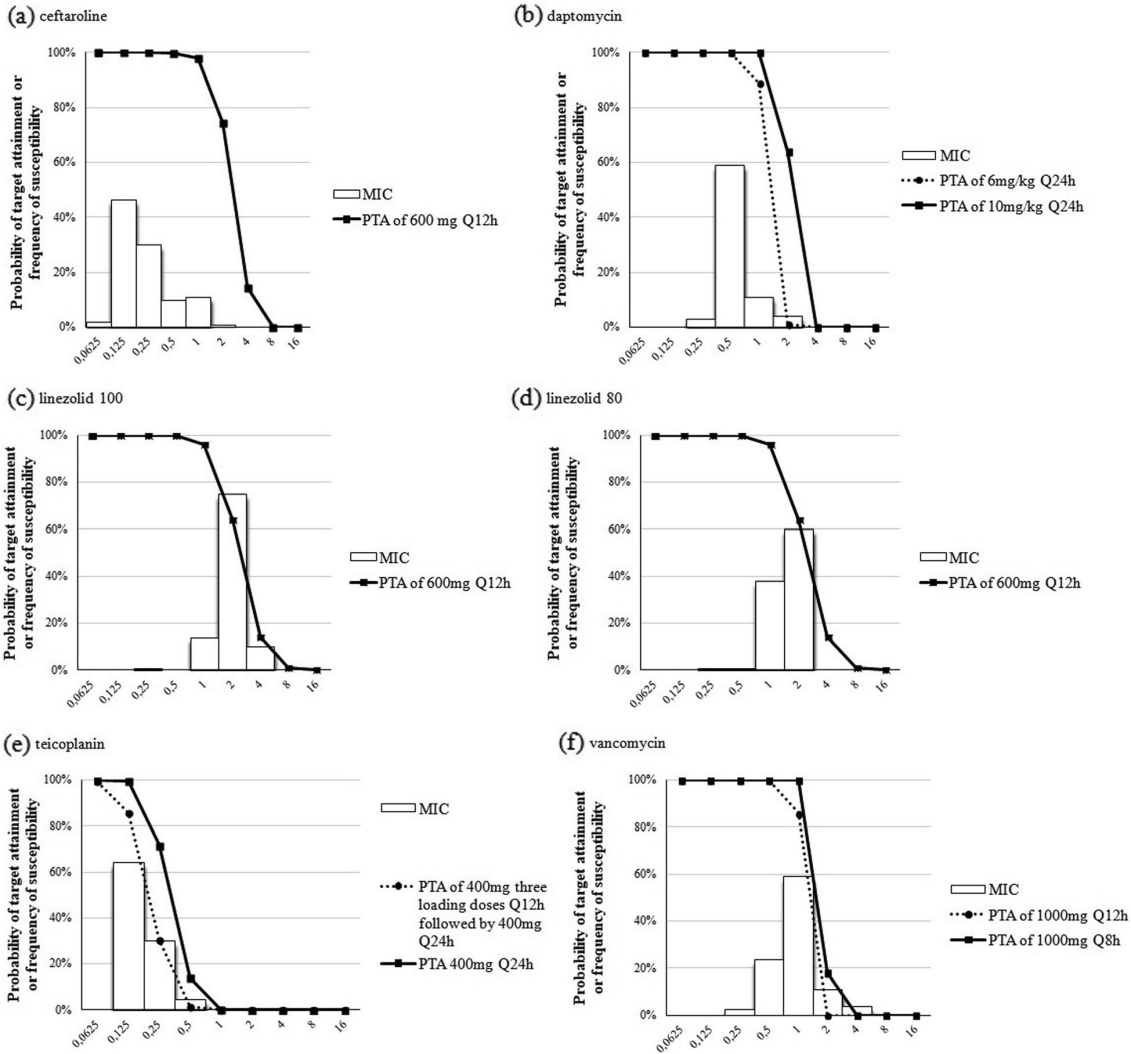
Probability of target attainment as a function of increasing minimum inhibitory concentration (MIC) Lines represent PTA of each dose regimen. The MIC distributions in the plots show the frequency of isolates susceptibility. * The MIC values for linezolid were read at the first well where the trailing begins without regard for pinpoint trailing, as CLSI M100-S26 instructions, being designated as linezolid-80; and at 100% inhibition of growth, being designated as linezolid-100. (**a**) Probability of ceftaroline achieving a *f*T MIC > 51%; (**b**) Probability of daptomycin achieving a total AUC/MIC > 40; (**c**) and (**d**) Probability of linezolid 100 and linezolid 80 achieving a total AUC/MIC > 82.9; (**e**) Probability of teicoplanin achieving a total AUC/MIC > 900; (**f**) Probability of vancomycin achieving a total AUC/MIC > 350.

Fig. 1 summarizes the MIC distributions and PTAs for ceftaroline, daptomycin, linezolid-100, linezolid-80, teicoplanin and vancomycin. Linezolid, vancomycin and all daptomycin regimens achieved > 90% target attainment up to MICs of 1 mg/ L. It was observed > 90% PTA up to MIC of 0.125 and 0.25 mg/ L for teicoplanin three 400 mg q12h followed by 400 mg q24h and for teicoplanin 400 mg q12h, respectively.

Table 2 summarizes the CFRs against the entire collection of BSI *S. aureus* isolates. Ceftaroline (98%) and a higher daptomycin dose regimen (10 mg/kg) (92%) both achieved the CFR threshold defined as a limit for optimal therapy against the population tested. Teicoplanin 400 mg q12h provided 86% CFR, while daptomycin 6 mg/kg, vancomycin 1000 mg q8h and 1000 mg q12h, and linezolid 600 mg q12h all provided below 80% CFRs.

**Table 2:** Cumulative fraction of responses (CFR) against 110 *Staphylococcus aureus,* including MRSA, from bloodstream infections

Antimicrobial	Regimen	CFR	Sensitivity Analysis
Ceftaroline	600 mg q12h	98%	96–99%
Daptomycin	10 mg/kg q24h	92%	21–100%
Teicoplanin	400 mg q12h	86%	44–78%
Daptomycin	6 mg/kg q24h	79%	3–99%
Linezolid-80^a^	600 mg q12h	77%	51–94%
Vancomycin	1000 mg q8h	76%	71–77%
Teicoplanin	three 400 mg loading doses q12h followed by 400 mg q24h	65%	8–29%
Linezolid-100^a^	600 mg q12h	64%	34–87%
Vancomycin	1000 mg q12h	62%	40–62%

^a^: *MIC* endpoint values for linezolid were read at the first well where the trailing begins without regard for pinpoint trailing, as *CLSI M100-S26* instructions, being designated as linezolid-80; and at 100% inhibition of growth, being designated as linezolid-100

Table 2 also shows the sensitivity analyses for the PD targets used against the entire BSI *S. aureus* isolates. Ceftaroline achieved the greatest CFR and remained above optimal threshold (≥ 90%) despite different targets used. A very wide range of CFRs for daptomycin minimum bacteriostatic and minimum bactericidal targets (a CFR between 2 and 99% and 15–100% for 6 mg/kg and 10 mg/kg regimens, respectively) were observed. Teicoplanin trough concentration values targets provided inferior CFR results when compared to the clinical derived PK target. Sensitivity analysis evidenced that optimal CFR for linezolid was not achieved even if the lowest PD was chosen (AUC/MIC of 51.85). As indicated in Table 2, CFR were similar between AUC/MIC > 350 (73%) and AUC/MIC > 400 (76%) targets for vancomycin 1000 mg q8h regimen; considering 1000 mg q12h regimen, however, a large variation (40 and 62%, respectively) was reported.

## Discussion

Inappropriate antibiotic therapy is identified as an important predictor of mortality among patients with *S. aureus* bacteremia and higher vancomycin MIC values may be predictive of treatment failure [6, 36]. Given the availability of different antimicrobials for the treatment of nosocomial *S. aureus* infections, define empirical options for BSI in settings where high-vancomycin MIC *S. aureus* isolates are prevalen needs further discussion. The present study analyzed 110 single and consecutive *S. aureus* isolates associated to unique BSI episodes, obtained during 2011 to 2013, from a complex hospital environment in Brazil. Our current *S. aureus* sample represents this scenario, since 28% of all presented vancomycin MIC ≥2 mg/L. In this environment, both vancomycin regimens (1000 mg q12h and 1000 mg q8h) were unable to achieve the optimal defined threshold CFR (62 and 76%, respectively), probably due to a high MIC prevalent environment. As confirmed by the sensitivity analysis, even higher vancomycin dose regimens would still attain suboptimal efficacy considering both PD (clinical cure and bacterial eradication) indexes.

Determining treatment strategies using other anti-staphylococcal agents should be explored in order to provide alternative empiric treatment options in settings where elevated vancomycin MIC are prevalent [22, 37]. Based in our model, higher daptomycin dosing regimen (10 mg/kg q24h) and currently FDA approved ceftaroline dosing regimen (600 mg q12h) performed an interesting therapeutic options from a PD standpoint. Ceftaroline is the only beta-lactam antibiotic commercially available in many countries (USA and others) with inherent activity against MRSA, however it is not currently approved for *S. aureus* BSI treatment [38]. On the other hand, daptomycin has label indication for the treatment of *S. aureus* BSI and right-side endocarditis [9].

Historically, daptomycin has been used as salvage therapy in patients failing vancomycin therapy, but its use has been increasingly common as initial empiric therapy [39]. Although daptomycin FDA approved dose regimen for of *S. aureus* BSI treatment is 6 mg/kg q24h, higher dose regimens would probably need to be used (10 mg/kg q24h or more) in order to overcome daptomycin non-susceptibility or in specific situations such as ﻿complicated or persistent MRSA bacteremia or profound infections [38]. Notably, there is an apparent correlation between daptomycin non-susceptibility and vancomycin intermediate resistance [40, 41].

As a limitation of the data, we must consider the sensitivity analysis for daptomycin. It illustrates the difficulties of translating in vitro bacteriostatic and bactericidal concepts into clinical practice [42]. In summary, bacteriostatic in vitro threshold used for daptomycin and observed in murine model studies would be only achieved with high-dose daptomycin regimens; thus, bactericidal targets would rarely be attained against isolates with similar MIC distributions. This also shows that the level of uncertainty in the current study is higher for daptomycin and further studies are needed to define relevant PD targets.

Ceftaroline is approved by the FDA for bacterial skin infections and community-acquired bacterial pneumonia, but there is increasing evidence of its use for treatment of patients with *S. aureus* BSI [8, 38, 40], including treatment of serious MRSA infections and those caused by strains with reduced susceptibility to vancomycin and non-susceptibility to daptomycin [43]. Although a *fT >* MIC between 25 to 30% is reported as an appropriate target for complicated skin and skin structure infections [26], we have chosen a more aggressive PD target (*fT >* MIC values of 51%) considering that BSI is a life-threatening infection and potentially higher exposures would be needed. Further randomized clinical trials are needed to confirm ceftaroline role as a viable treatment option against *S. aureus* BSI. However, observational studies demonstrate ceftaroline viability as potential treatment option for this condition [8, 38]. From the PD standpoint, even applying higher exposures targets ceftaroline performed well against the current *S. aureus* BSI isolates.

Teicoplanin has been widely reported as comparable to vancomycin in terms of efficacy and has been commonly prescribed in many parts of the world (excluding the USA) [44, 45]. Despite its availability in clinical practice for many years, the optimal PD profile is still under debate. Historically, trough concentrations have been utilized to characterize the adequacy of the glycopeptide PD profile; however, trough concentration alone is not an entirely appropriate target predictive of clinical success as sensitivity analysis demonstrated that even if a most aggressive trough concentration value was chosen (i.e., > 20 mg/L), the CFR observed in trough concentration targets was inferior to the PD value associated with successful outcome [34, 35, 46].

As a result of this discordance with trough value, we utilized a target AUC/MIC in the current study. Although a 400 mg once-daily regimen of teicoplanin is often utilized in clinical practice, our data suggests that a 400 mg q12h regimen will provide a more optimal PD profile. In agreement with our findings, a recent clinical study showed that teicoplanin maintenance dosing of 400 mg q12h for severe infections due to MRSA provided higher clinical response rates and lower BSI-related mortality rates [44].

In this study the linezolid regimen (600 mg q12h) presented a CFR of 77%, far below the threshold defined as optimal response. This is partially explained by an elevated prevalence (60%) of isolates with linezolid MIC ≥2 mg/L. These findings were supported by the sensitivity analysis, given the large variation observed between the alternative PD targets proposed and even if less conservative PD target (reflecting the minimum PD index associated to clinical cure) were chosen, higher CFR thresholds were not achieved. Considering off-label use of linezolid for a potentially severe infection can be applied but other treatment options should be considered if isolates with linezolid MIC ≥2 mg/L are locally prevalent.

Only antimicrobials used for treatment of MRSA infections were accessed in the current study, although there are other available therapies not addressed in the current evaluation. Empirical therapy of *S. aureus* infections in hospital settings with elevated MRSA prevalence must consider a complex MIC distribution pattern and, consequently, complex drug exposure issues. A different scenario should be expected in environments with prevalent methicillin susceptible *S. aureus*.

Among the limitations of the present study, the selection of PK published studies included data derived from different clinical indications given the lack of comparable PK trials for each of these agents in the same patient population. On the other hand, we also avoided the inclusion of PK data derived exclusively from critically ill patients, due to the vast array of pathophysiological changes which affects antibiotic dosing in the critically ill [11]. Another potential limitation is that the results of any simulation are dependent on local MIC distributions and may vary among periods and institutions.

Finally, environments where *S. aureus* vancomycin MIC ≥2 μg/mL are prevalent may have concerns about vancomycin lack of activity, particularly for difficult to treat infections. Thus, stochastic methods can be used to guide antimicrobial stewardship in situations where several therapeutic options are currently available.

## Conclusions

In conclusion, our study presents insights about alternative treatment strategies in setting where *S. aureus* vancomycin MIC is elevated (particularly ≥2 μg/mL). In this scenario, ceftaroline and high dose daptomycin (10 mg/kg q24h) regimens achieved CFRs ≥90%, whilst teicoplanin high dose (400 mg q12 hours) regimen achieved better CFR when compared to the most effective vancomycin regimen, although below the defined optimal threshold.

## Abbreviations

AUC: Area under the concentration time curve
BSI: Bloodstream infections
CFR: Cumulative fraction of response
CFU: Colony-forming unit
CLSI: Clinical & laboratory standards institute
*f*AUC: Unbound drug fraction area under the concentration time curve
FDA: Food and drug administration
fT > MIC: Unbound drug fraction above minimum inhibitory concentrations
MIC: Minimum inhibitory concentrations
MRSA: Methicillin-resistant *S. aureus*
PD: Pharmacodynamic
PK: Pharmacokinetic
PTA: Probability of target attainment
VISA: Vancomycin intermediate *S. aureus*
